# Oxidative Stress: Pathological Driver in Chronic Neurodegenerative Diseases

**DOI:** 10.3390/antiox14060696

**Published:** 2025-06-09

**Authors:** Zhao Zhong Chong, Nizar Souayah

**Affiliations:** 1 Department of Neurology, New Jersey Medical School, Rutgers University, 185 S Orange, Newark, NJ 07103, USA; 2 Department of Neurology, New Jersey Medical School, Rutgers University, 90 Bergen Street DOC 8100, Newark, NJ 07101, USA

**Keywords:** oxidative stress, mitochondria, nicotinamide adenine dinucleotide phosphate, Alzheimer’s disease, Parkinson’s disease, amyotrophic lateral sclerosis

## Abstract

Oxidative stress has become a common impetus of various diseases, including neurodegenerative diseases. This review introduces the generation of reactive oxygen species (ROSs) in the nervous system, the cellular oxidative damage, and the high sensitivity of the brain to ROSs. The literature review focuses on the roles of oxidative stress in neurodegenerative diseases, including Alzheimer’s disease (AD), Parkinson’s disease (PD), Huntington’s disease (HD), and amyotrophic lateral sclerosis (ALS). Oxidative stress occurs when excessively produced free radicals are beyond the capability of endogenous antioxidants to scavenge, leading to the oxidation of proteins, lipids, and nucleic acids, stimulating neuroinflammatory responses, causing neuronal dysfunction, senescence, and death. The dysfunctional mitochondria and aberrant activities of metabolic enzymes are the major source of ROSs. The high vulnerability of the nervous system to ROSs underlies the critical roles of oxidative stress in neurodegenerative diseases. Gene mutations and other risk factors promote the generation of ROSs, which have been considered a crucial force causing the main pathological features of AD, PD, HD, and ALS. As a result, antioxidants hold therapeutic potential in these neurodegenerative diseases. The elucidation of the pathogenic mechanisms of oxidative stress will facilitate the development of antioxidants for the treatment of these diseases.

## 1. Introduction

Oxidative stress has become a common pathogenic contributor to the development and progression of various diseases, including cardiovascular disease, cancer, diabetes, and acute and chronic neurodegenerative disorders [1,2]. Oxidative stress occurs when oxidants and antioxidants lose balance in the body [3]. In other words, oxidative stress happens when free radicals are generated in excess, which is beyond the capability of endogenous antioxidants to scavenge. Since its conceptualization, oxidative stress has been extensively investigated in terms of its roles and pathogenic mechanisms in various diseases. Reactive oxygen species (ROSs) and reactive nitrogen species are generally considered prominent oxidants.

### 1.1. Endogenous Oxidants and Antioxidants

ROSs primarily consist of superoxide anion radicals, hydrogen peroxide, singlet oxygen, and hydroxyl radicals. Reactive nitrogen species are nitrogen-based radicals, including nitric oxide (NO), peroxynitrite, and nitrogen dioxide. Among ROSs, superoxide radical is the most common oxygen-free radical. Hydroxyl radical is the most active and potent oxygen radical. Hydroxyl radical is produced by the interaction between superoxide and hydrogen peroxide, catalyzed by ferrous iron (Haber–Weiss reaction). Superoxide radical interacts with NO to form peroxynitrite, a highly toxic species, and further form peroxynitrous acid, which can undergo spontaneous decomposition to form hydroxyl radical. In addition, many other active species and intermediates may also play an important role in the induction of oxidative stress [4].

The endogenous antioxidant system includes, but is not limited to, superoxide dismutase (SOD), catalase, and glutathione peroxidase (GPX)—SOD functions to catalyze the conversion of superoxide into hydrogen peroxide and oxygen. Three main SODs have been identified with different distribution. SOD1 is Cu, Zn-SOD that is expressed in the cytosol; SOD2 is Mn-SOD found in mitochondria; while SOD3 is extracellular Cu, Zn-SOD [5]. GPX reduces hydrogen peroxide and other hydroperoxides, and at least eight GPXs have been identified. GPX1 is a ubiquitous GPX that is expressed intracellularly and functions to reduce hydrogen peroxide to water and has the property of peroxynitrite reductase. GPX1 has been found in mitochondria, nucleus, and cytosol, and its expression was identified in both neurons and glial cells [6]. The expression of GPX2 is primarily found in the intestinal epithelium. GPX3 is an extracellular one; while the expression of GPX4 is found in most tissues, particularly in testes, its expression functions to protect sperm against oxidative stress. Vitamins C and E, obtained through dietary intake, are both powerful antioxidants. However, vitamin C (ascorbic acid) is different in that it is a water-soluble vitamin, and vitamin E (alpha-tocopherol) is lipid-soluble. The lipid-insolubility limits the access of vitamin C across the blood–brain barrier into the brain.

Under physiological conditions, ROSs are produced at low levels and balanced by endogenous antioxidant systems. Either overproduction of ROSs, reduced antioxidant capacity, or both under pathological conditions leads to oxidative stress. In most cases, the induction of oxidative stress is ascribed to the overproduction of oxidant species.

### 1.2. ROS Generation

**Figure 1:**
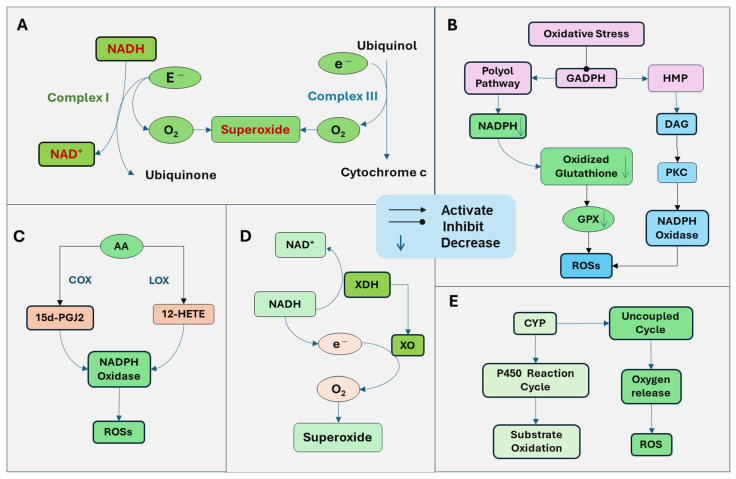
Generation of reactive oxygen species (ROSs) through several sources. (**A**) Mitochondrial respiration with the electron transport chain produces ATP. Simultaneously, ROSs are produced as a by-product. Complex I, nicotinamide adenine dinucleotide (NADH) dehydrogenase oxidizes NADH and transfers electrons to ubiquinone. When electrons are transferred to oxygen in impaired mitochondria, it leads to oxygen reduction and superoxide formation. Complex III, the cytochrome bc1 complex, transfers electrons from ubiquinol to cytochrome C and can also transfer an electron to oxygen to produce superoxide. (**B**) Oxidative stress inhibits the activity of glyceraldehyde-3-phosphate dehydrogenase (GAPDH), the key enzyme of glycolysis, leading to the activation of the polyol and hexose monophosphate (HMP) pathways, resulting in the activation of protein kinase C (PKC) and increased production of ROSs. The polyol pathway uses nicotinamide adenine dinucleotide phosphate (NADPH), leading to the depletion of NADPH and subsequent downregulation of glutathione reductase. Thus, oxidized glutathione cannot be reduced, which is essential for GPX. The ability of GPX to reduce hydrogen peroxide is attenuated. In addition, the increase in the polyol pathway and HMP pathway leads to an increased production of glycolytic precursor diacylglycerol (DAG). DAG and PKC trigger the production of ROS by activating NADPH oxidase. (**C**) Arachidonic acid (AA) is released from the membrane lipid and is metabolized by lipoxygenases (LOX) and cyclooxygenases (COX), the products of which stimulate NADH oxidase. NADPH oxidase produces superoxide by catalyzing the transfer of an electron from NADPH to oxygen. The COX terminal metabolite, the 15-deoxy-Delta (12, 14)-prostaglandin J_2_ (15d-PGJ_2_) promotes the ROSs. The 12-LOX product, 12-hydroxyeicosatetraenoic acid (12-HETE), can stimulate NADH oxidase to increase ROS production. (**D**) Xanthine dehydrogenase (XDH) is an enzyme that functions in the metabolism of purines. It oxidizes hypoxanthine to xanthine and then to uric acid. Under pathological conditions, xanthine dehydrogenase is converted to xanthine oxidase (XO), which cannot utilize NADH as a donor and NAD^+^ as an electron acceptor but radically reduces oxygen to superoxide radical and hydrogen peroxide. (**E**) The cytochrome P450 (CYP) enzymes function through the CYP reaction cycle, in which oxygen is utilized to oxidize substrates, and sometimes, an uncoupling process can happen, failing in oxygen transfer to the substrate and generation of ROSs.

The generation of ROSs is mediated by multiple mechanisms (Figure 1). The mitochondria are the primary source of cellular energy through oxidative phosphorylation on the electron transport chain system. Through mitochondrial respiration with the electron transfer, ATP is generated by a series of reactions across the mitochondrial respiration complexes (I-V). Simultaneously, ROSs are produced as by-products. Complex I, nicotinamide adenine dinucleotide (NADH) dehydrogenase, is the first enzyme in the electron transport chain. Complex I oxidizes NADH and transfers electrons to ubiquinone. ROSs are generated when electrons are transferred to oxygen in impaired mitochondria, leading to oxygen reduction and superoxide formation [7]. Complex III, the cytochrome bc1 complex, transfers electrons from ubiquinol to cytochrome C. Electrons are transferred one at a time to form a ubisemiquinone radical, which can transfer an electron to oxygen, generating superoxide (Figure 1A). The predominantly produced ROS through Complex I and Complex III is superoxide, with less hydrogen peroxide formed [7]. The superoxide radical is turned into hydrogen peroxide by mitochondrial dismutase SOD.

The activation of NADPH oxidase is also an important source of ROSs. Increased ROSs and oxidative stress induction can induce pathological conditions that promote the NADPH oxidase and the generation of ROSs. For example, oxidative stress inhibits the activity of glyceraldehyde-3-phosphate dehydrogenase (GAPDH), the key enzyme of glycolysis. Under this condition, glucose products are metabolized through the polyol and hexose monophosphate (HMP) pathways instead of glycolysis, resulting in the activation of protein kinase C (PKC) and increased production of ROSs. The polyol pathway uses nicotinamide adenine dinucleotide phosphate (NADPH), leading to the depletion of NADPH and subsequent downregulation of glutathione reductase. Thus, oxidized glutathione cannot be reduced, which is essential for GPX. Consequently, the ability of GPX to reduce hydrogen peroxide is diminished. In addition, the increased polyol pathway leads to increased production of diacylglycerol (DAG). The HMP pathway also increases the production of DAG and activation of PKC. DAG triggers the production of ROS by activating NADPH oxidase [8]. PKC has been demonstrated to activate NADPH oxidase [9]. NADPH oxidase on the plasma membrane is one of the major sources of ROSs in cells (Figure 1B).

The metabolites of arachidonic acid (AA) by lipoxygenases (LOX) and cyclooxygenases (COX) have been demonstrated to stimulate NADPH oxidase [10]. NADPH oxidase produces superoxide by catalyzing the transfer of an electron from NADPH to oxygen. Cytosolic phospholipase A2 (cPLA2) promotes the release of AA from membrane lipids. AA is further metabolized by LOX and COX to generate bioactive eicosanoids, including prostaglandins, thromboxane, and leukotrienes, which may stimulate NADPH oxidase. The COX terminal metabolite, the 15-deoxy-Delta (12, 14)-prostaglandin J_2_ (15d-PGJ_2_) has been shown to promote the ROSs [11]. LOX is assigned as 5-, 8-, 12-, and 15-LOX, depending on the insertion position of oxygen in AA. The 12-LOX product, 12-Hydroxyeicosatetraenoic acid (12-HETE), can stimulate NADPH oxidase to increase ROS production [12] (Figure 1C).

Xanthine dehydrogenase is an enzyme that functions in the metabolism of purines. It oxidizes hypoxanthine to xanthine and then to uric acid. Under pathological conditions, xanthine dehydrogenase is converted to xanthine oxidase, which cannot utilize NADH as a donor and NAD^+^ as an electron acceptor, radically reducing oxygen to superoxide radical and hydrogen peroxide [13] (Figure 1D).

The cytochrome P450 (CYP) enzymes play a crucial role in the drug and xenobiotic metabolisms, cholesterol synthesis, and detoxification of foreign chemicals. The function of CYP is mediated through the CYP reaction cycle, in which oxygen is utilized to oxidize substrates, and sometimes, an uncoupling process can happen, failing in oxygen transfer to the substrate and generation of ROSs [14] (Figure 1E).

### 1.3. Oxidative Damage

Excessive ROSs attack various cellular components, including proteins, lipids, DNA, and other molecules. Lipid oxidation induced by oxidative stress leads to lipid peroxidation. The key lipid targets of ROSs are unsaturated fatty acids. ROSs initially attack membrane lipids, producing lipid radicals, which subsequently form highly reactive lipid peroxy radicals, damaging the membrane lipids. Lipid peroxidation produces toxic molecules that result in membrane damage, an inflammatory response, and cell degeneration [15,16,17]. Common stable lipid peroxidation products include malondialdehyde (MDA), 4-hydroxynonenal (HNE), isoprostanes, and acrolein, which can be used as biomarkers of oxidative stress.

MDA is the product of the interaction between free radicals and polyunsaturated fatty acids in cell membranes. The levels of MDA in blood or tissues can be used to evaluate lipid peroxidation and oxidative stress. It is also highly toxic and interacts with and damages proteins and DNA [18]. MDA-related carbonyl stress has been demonstrated to increase intracellular Ca^2+^, which damages rat hippocampal neurons by triggering Ca^2+^ influx and motivating Ca^2+^ release from intracellular stores [19]. MDA increased the ratio of γ-aminobutyric acid to glutamate, impairing the homeostasis between excitatory and inhibitory neurons and downregulating synaptic function [20].

HNE has been implicated in neuron death signaling pathways. Elevated HNE levels in the brain and cerebrospinal fluid have been observed in patients with neurodegenerative diseases [21]. HNE can bind to cellular proteins, disrupting their structure and function, and causing neuronal cell death. HNE has been demonstrated to damage mitochondria, leading to the impairment of energy production and increased generation of ROS. HNE also increased the expression of mitochondrial proapoptotic protein Bax and p53 in neuroblastoma cell lines and apoptotic activation of caspase-3 [22]. Moreover, HNE can interfere with neurite outgrowth and neuronal microtubule architecture [23].

F2-isoprostanes and neuroprostanes are the oxidative products of arachidonic acid and docosahexaenoic acid, respectively; both are abundant polyunsaturated fatty acids in the brain. Elevated levels of isoprostanes have been found in the brains and cerebrospinal fluid of patients with multiple neurodegenerative diseases [24]. Their roles in neurodegeneration remain to be investigated. Acrolein is a pro-oxidative aldehyde that initiates oxidative stress, induces protein aggregation, and disrupts synaptic function [25].

Protein oxidation may cause protein carbonylation, amino acid oxidation, the cleavage of peptide bonds, and protein aggregation and degradation, leading to dysfunction of proteins and induction of cell death [17,26]. Free radicals target lysine, arginine, and proline on amino acid side chains or the protein backbone for oxidation. Carbonyls, such as aldehydes and ketones, are highly active and can interact with proteins, lipids, and DNA, disrupting cellular processes, damaging neurons, which has been associated with age-related diseases [27]. Oxidative stress also induces the glycation of proteins to form advanced glycation end products (AGEs). AGEs can induce inflammatory responses by activating a series of cell signaling pathways that regulate the release of inflammatory cytokines [28]. Lipid peroxidation products, such as HNE and MDA, can react with proteins to form adducts on amino acid residues (lysine, histidine, and arginine). The resulting advanced lipid peroxidation end products (ALEs) and AGEs, which can aggravate the oxidative cascade and induce inflammatory responses, have been closely implicated in aging and neurodegenerative diseases [29].

ROSs also attack DNA bases, causing strand breaks, altering DNA base structure with the production of 8-hydroxy-2′-deoxyguanosine (8-OHdG), disrupting DNA repair mechanisms, and DNA-protein cross-links, leading to genomic dysfunction, neuronal death, and possible mutagenesis [30].

The oxidation products of lipid, protein, and DNA can function as biomarkers for monitoring oxidative stress, disease progression, and treatment outcomes in various diseases, including neurodegenerative diseases. They may also be targeted for therapeutic purposes in oxidation-associated pathological conditions.

## 2. The Vulnerability of the CNS to Oxidative Stress

The nervous system is highly sensitive to oxidative stress. Both peripheral nerves and the central nervous system (CNS) are rich in lipids and mitochondria, which are vulnerable targets of oxidative damage [31]. The brain is the highest oxygen-demanding organ in the body, digesting 20% of the oxygen, the process of which enhances ROS generation [2,32]. The high energy demand of the brain depends more heavily on mitochondria, and more ROSs will be produced with more active mitochondrial activity. In addition, the enrichment of unsaturated fatty acids in the CNS facilitates the attack by oxygen-free radicals. Moreover, neurons are nondividing cells, meaning that damaged neurons by oxidative stress lack replacement, making the neuronal damage irreversible. Other underly mechanisms have been elucidated previously [33,34]. Besides its sensitivity to oxidative stress, the brain has a low antioxidant system, which also lowers the threshold of oxidative stress. Much lower catalase levels in neurons and GSH activity have been observed; 50 times lower levels of catalase are in neurons than in hepatocytes [14,35].

The generation of ROSs in the brain regions is different [36]. The glial cells (microglia and astrocytes) and neurons can all be sources of ROS in the CNS. Under physiological conditions, the highest ROS production derived from glial cells is found in the brain stem and cerebellum. Glutamate and ATP can stimulate glial cells and neurons to produce ROSs with maximal rates of ROS generation in the midbrain, which has the lowest GSH level and a higher lipid peroxidation level.

## 3. Crosstalk Between Oxidative Stress and Inflammation

Oxidative stress and inflammation interact with and stimulate each other, expanding the cascade reaction in the pathological process. ROSs induce cellular damage, producing toxic components and triggering the activation of an inflammatory response, while inflammatory cytokines and chemokines recruit immune cells and inflammatory cells, including macrophages and neutrophils, to the inflammatory site, releasing more cytokines and ROSs, aggravating oxidative stress. In the CNS, microglia and astrocytes play a critical role in the inflammatory response and in the generation of ROSs.

### 3.1. Roles of Microglia

ROSs may contribute to the induction of inflammation by activating microglia, macrophage-like cells and sentinel cells in the CNS. Under physiological conditions in the adult brain, microglia typically exist in a resting state and are activated in response to brain injury, releasing free radicals and inflammatory cytokines and causing brain tissue damage.

Activated microglia-produced ROSs are mainly superoxide radicals. One major source of ROSs in microglia is through activating NADPH oxidase [37]. Microglial NADPH oxidase contains the catalytic subunit NOX2 (gp91phox). To date, at least seven human homologs of the catalytic subunit of the phagocyte NADPH oxidase have been found: NOX1, NOX3, NOX4, NOX5, dual oxidase (DUOX)1, and DUOX2. NADPH oxidases with different catalytic subunits differ in their subcellular localization and implications in pathophysiological conditions. NOX2 is the first identified NOX. NADPH oxidase in microglia/macrophages constitutively expresses NOX2, with much lower expression of NOX4 [38].

In response to CNS injury insult, microglia are activated and produce superoxide, which may reflect initial defense for the protection of the CNS but induce an inflammatory reaction, damaging the brain tissues [37]. Amyloid-β1-42 (Aβ_42_), the extracellular deposition of which contributes to the formation of amyloid fibril plaques, can significantly increase ROSs, NOX2 expression, and interleukin 1β (IL-1β) secretion in microglia [39]. Neurons may also be the source of ROSs by NADPH oxidase. Intraperitoneal lipopolysaccharide (LPS) injection led to persistent upregulation of NOX2 in both microglia and neurons in mice of a chronic mouse model of Parkinson’s disease (PD) with increased ROS production, lipid peroxidation, and degeneration of dopaminergic neurons [40]. Inhibition of NADPH oxidase polarized microglia from an inflammatory phenotype (M1) to an anti-inflammatory phenotype (M2) [41], suggesting that generation of ROSs by NADPH oxidase induces inflammatory responses.

Mitochondria are the primary source of ROSs in microglia. In microglia, extracellular copper at sub-neurotoxic concentrations can induce ROS generation, which is independent of NADPH-oxidase. However, copper treatment stimulates a rapid release of hydrogen peroxide-paralleled mitochondrial superoxide production from microglial cells. In addition, copper-induced release of TNF-α and NO from microglia was demonstrated to initiate microglia-mediated neurotoxicity [42]. The activation of microglia promotes the reverse electron transport and the production of ROS through mitochondrial complex I activity. The blockade of complex I-activated microglia protects against inflammatory injury of the CNS [43].

### 3.2. Roles of Astrocytes

In the CNS, astrocytes play a crucial role in maintaining the normal function of the brain, playing important roles in nourishing neurons, maintaining the integrity of the blood–brain barrier, regulating synapse activity, and processing cell metabolites. Astrocytes are the primary source of antioxidants for neutralizing ROSs to protect the CNS against oxidative stress. However, under pathological conditions, astrocytes may become one of the main sources of ROSs to activate microglia or damage neurons.

The generation of ROSs in astrocytes comes from several sources. Mitochondria play a crucial role in astrocytic redox regulation under physiological or pathological conditions. A significant portion of ROS generated by astrocytes is from the mitochondria. Superoxide production from mitochondria has been associated with the apoptotic process of motor neurons [44]. Overexpression of antioxidative genes, MnSOD and GPX4, in mitochondria significantly increased cell survival in mutant SOD1 motor neurons [45]. Astrocytes also express a low level of NADPH oxidase, which progressively increases with aging [46]. In addition, astrocytes express NO synthase (NOS) and are the source of NO and related free radicals. There are three NOSs: neuronal NOS (nNOS), endothelial NOS (eNOS), and inducible NOS (iNOS), which are all found in astrocytes. Inflammatory cytokines have been demonstrated to activate astrocytic iNOS [47] and NO released from astrocytes has been implicated in the astrocyte-induced neuronal degeneration [48].

### 3.3. Cell Signaling Pathways

**Figure 2:**
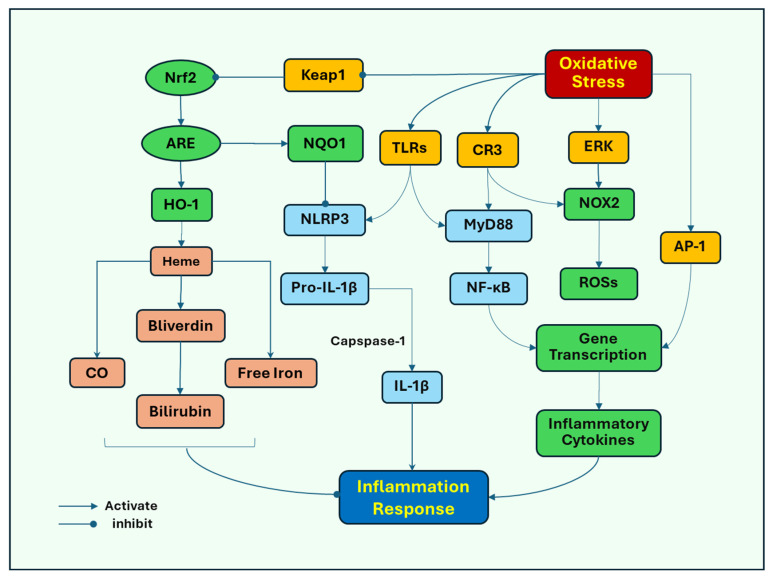
Oxidative stress mediates cell signaling pathways that lead to neuroinflammation. Oxidative stress induces neuronal damage, releasing endogenous damage-associated molecular patterns (DAMPs), which activate microglial Toll-like receptors (TLRs) to increase the activity of NOD-like receptor protein 3 (NLRP3) inflammasome, and extracellular signal-regulated kinase (ERK), leading to inflammatory responses and subsequent activation of NOX2. Oxidative stress activates NLRP3 to promote the release of the proinflammatory cytokine IL-1β in its pro-form, which is subsequently cleaved by caspase-1 into its mature form. Oxidative stress can also lead to increased expression of complement receptor 3 (CR3). Either TLRs or CR3 recruit myeloid differentiation primary response protein 88 (MyD88), to activate nuclear factor kappa-light-chain-enhancer of activated B cells (NF-κB), which redistributes from the cytosol to the nucleus, where it promotes the transcription of proinflammatory cytokine genes. In addition, oxidative stress activates AP-1 (activator protein 1), which binds to inflammatory genes to promote the gene transcription of cytokines, chemokines, and adhesion molecules. In response to oxidative stress, nuclear factor erythroid 2-related factor 2 (Nrf2) is released from the binding to its inhibitory protein, Kelch ECH-associated protein 1 (Keap1) and translocated to the nucleus where it binds to antioxidant response element (ARE), triggering the gene expression of antioxidants, including heme oxygenase-1 (HO-1). HO-1 breaks down heme into carbon monoxide (CO), free iron, and biliverdin, then to bilirubin, which has anti-inflammatory effects. Nrf2 also induces the expression of quinone oxidoreductase (NQO1), inhibiting the activation of NLRP3 inflammasome.

Several signaling pathways induced by oxidative stress have been proposed to stimulate inflammatory responses. Toll-like receptors (TLRs) play an important role in response to oxidative stress-induced microglial activation as the major pattern recognition receptors (PRRs) that identify pathogen-associated molecular patterns (PAMPs) and endogenous damage-associated molecular patterns (DAMPs). Oxidative stress induces neuronal damage, releasing DAMPs, which act on microglial TLR4 to mediate the activation of NOD-like receptor protein 3 (NLRP3) inflammasome, nuclear factor kappa-light-chain-enhancer of activated B cells (NF-κB), and mitogen-activated protein kinases (MAPKs), such as extracellular signal-regulated kinase (ERK) [49], leading to inflammatory responses and subsequent activation of NOX2. Oxidative stress activates NLRP3 to promote the release of the proinflammatory cytokines IL-1β and IL-18 as their pro-forms, which are subsequently cleaved by caspase-1 into their mature forms [50]. Oxidative stress and its associated damage induce translocation of NF-κB from the cytosol to the nucleus, where it promotes the transcription of proinflammatory cytokine genes, such as TNF-α and iNOS, and subsequently increases the release of TNF-α and NO [51]. ERK can also promote the nuclear translocation of NF-κB to initiate the release of inflammatory cytokines (Figure 2).

Complement receptor 3 (CR3), another PRR, mediates microglial NOX2 activation and dopaminergic neurodegeneration [52]. Oxidative stress can lead to increased expression of CR3, which induces the recruitment of immune cells to the injury sites, and inflammatory tissue damage has also been implicated in acute brain injury [53]. Either TLRs or CR3 recruit cytoplasmic TIR domain-containing adaptor proteins, such as myeloid differentiation primary response protein 88 (MyD88), to activate NF-κB [54] and subsequent induction of proinflammatory responses.

Activator protein 1 (AP-1) has been closely associated with regulating inflammatory responses and oxidative stress. AP-1 is a transcription factor comprising heterodimeric complex formed by the dimerization of a characteristic basic-region leucine zipper domain in the Fos and Jun subunits. Oxygen radicals can induce the activation of AP-1 [55], which binds to inflammatory genes to promote the transcription of cytokine genes, including cytokines, chemokines, and adhesion molecules [56,57]. The activation of AP-1 has been associated with MAPKs and NF-κB. Activated MAPKs, in response to oxidative stress, induce the transcription of Fos and Jun genes, increasing the expression of the AP-1 complex [58]. NF-κB and AP-1 can interact with each other and NF-κB has been found to enhance the expression of c-Fos and AP-1 activity by mediating the expression level of Elk-1, a transcription factor that regulates genes involved in cell growth, differentiation, and survival [59].

Nuclear factor erythroid 2-related factor 2 (Nrf2) may represent a cell defense mechanism against oxidative stress. Nrf2 is a transcription factor for an antioxidant response. Nrf2 promotes the expression of antioxidant genes by binding to the antioxidant response element (ARE). In response to oxidative stress, Nrf2 is released from its inhibitory protein, Kelch ECH-associated protein 1 (Keap1), and translocated to the nucleus where it binds to ARE, triggering the expression of antioxidant genes. Nrf2-ARE target several antioxidant enzymes, such as SOD, GPX, and catalase. The system also upregulates the detoxifying proteins and proteasomal degradation proteins that assist in removing damaged cellular components [60]. Heme oxygenase-1 (HO-1) is one of its targets of detoxifying enzymes Nrf2 induces the expression of HO-1 [61], which breaks down heme, a proinflammatory protein, into carbon monoxide (CO), free iron, and biliverdin then to bilirubin, which has anti-inflammatory effects [62]. Nrf2 also induces the expression of quinone oxidoreductase (NQO1), inhibiting the activation of NLRP3 inflammasome [50,63]. In addition, Nrf2 inhibits NF-ĸB-dependent transcription of inflammatory cytokine genes [64].

### 3.4. Feedback on Inflammation

As discussed above, oxidative stress is a critical inducer of inflammatory pathways; however, inflammation in cells also promotes the production of ROSs. Inflammatory cytokines such as TNF-α, IL-1β, and IL-6 can promote the activation of NADPH oxidase with the generation of ROS. The upregulation of NADPH oxidase has been observed in the brain with traumatic brain injury [65]. Activation of cytokine signaling pathways, including NF-κB and MAPKs, can also promote ROS production through activating NADPH oxidase, mitochondria, and xanthine oxidase [66]. Cytokines can inhibit the activity of antioxidant enzymes by downregulating the expression of antioxidant enzymes, such as SOD, catalase, and GPX, leading to an accumulation of ROSs [67].

### 3.5. Summary

The generation of ROSs and induction of oxidative stress in the brain can directly target neuronal cellular components and trigger inflammatory responses that contribute to neuronal damage. Inflammatory mediators also stimulate the generation of ROSs to aggravate oxidative stress. The interaction of oxidative stress and neuroinflammation causes a vicious cycle to damages components of cells, leading neurons to undergo degeneration and demise, which play pathogenic roles in neurodegenerative diseases.

Which one is the initiating factor to promote the progression of cellular injury? To clarify, the sequence may hold the key to determining the more effective therapeutic targets for the associated diseases. This sequence may be dependent on specific diseases and underlying causes. In case of neurodegenerative diseases, many risk factors have been shown to increase the generation of ROSs, and oxidative stress seems to occur first and subsequently activate the inflammatory cascade [68]. However, no solid evidence to exclude that neuroinflammation comes first to cause cellular damage and induce the generation of ROSs.

## 4. Role of Oxidative Stress in Alzheimer’s Disease

Alzheimer’s disease (AD) is the most common neurodegenerative disease among the older population, characterized by a progressive decline in cognitive function with memory loss. The two pathologic hallmarks of AD have been considered as extracellular plaques of β-amyloid peptide (Aβ) aggregates and intracellular neurofibrillary tangles composed of the hyperphosphorylated microtubular protein tau. Aβ is the cleavage product of β-amyloid precursor protein (APP) by sequential secretases. Both pathological changes have been associated with the induction of oxidative stress.

### 4.1. ROS Generation in AD

NADPH-oxidase-generated ROSs have been involved in AD. Elevated expression of NADPH oxidase in the frontal and temporal cortex postmortem brains of AD patients was observed [69]. Specific upregulation of NADPH oxidase expression and activity was found in the temporal gyri of mild cognitive impairment patients, not in late-stage AD patients, suggesting that NADPH oxidase-associated generation of ROS is implicated in the early pathogenesis of AD. In contrast, the application of apocynin to inhibit NADPH oxidase or genetic deletion of NOX2 in mice overexpressing mutant APP (Tg2576) attenuates oxidative stress and improves cerebrovascular function [70]. Deletion of NOX2 (gp91phox) also abolished oxidative stress and cerebrovascular dysfunction in transgenic mice overexpressing the APP [71].

Progressive mitochondrial impairment function has been considered the primary cause of ROS generation. Mitochondria are also a major target of oxidative stress, and ROSs aggravate the mitochondrial dysfunction. Mitochondrial function progressively declines with aging and AD, leading to decreased ATP production and increased generation of ROSs, and further impairing energy supply and mitochondrial function [72]. ROSs also impair mitochondrial biogenesis, attenuate mitophagy, and disrupt mitochondrial dynamics, leading to defects in mitochondrial fission and fusion [73]. Improving mitochondrial dynamics reduces superoxide levels in mitochondria [74]. Inhibition of mitochondrial respiration complex I and III by rotenone and antimycin increased the levels of ROSs and Aβ. Antioxidant treatment prevented mitochondrial dysfunction and diminished the formation of Aβ [75]. These studies explicitly indicate that mitochondrial-derived ROSs contribute to mitochondrial dysfunction and the pathogenesis of AD. APP and Aβ have also been found to be expressed in the mitochondria. The accumulation of Aβ within the mitochondrial matrix was observed, and Aβ can disturb the normal mitochondrial function, increase the generation of ROSs, and promote the permeability transition pore formation, leading to a mitochondrial cell death cascade [76]. In Tg2576 mice overexpressing APP, Aβ was found to elevate hydrogen peroxide levels since the levels were directly correlated with levels of soluble Aβ in Tg2576 mice. Cytochrome C oxidase activity was decreased in Tg2576 mice, suggesting that mutant APP and soluble Aβ impair the mitochondrial respiratory chain and possibly promote the progression of AD [77].

### 4.2. Risk Factors for AD and Oxidative Stress

**Figure 3:**
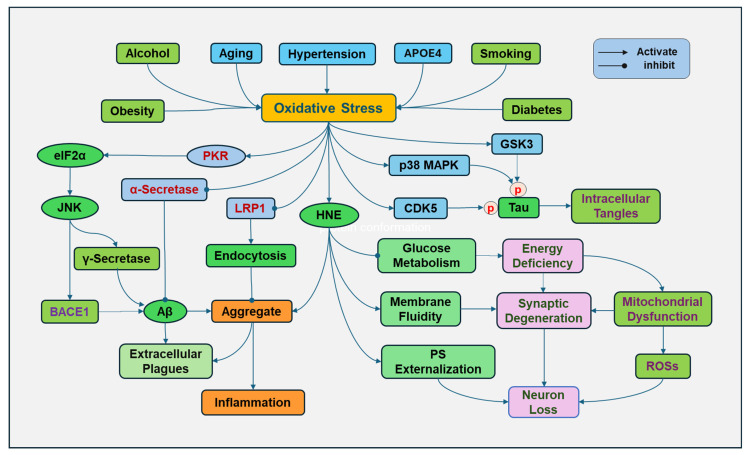
Oxidative stress in Alzheimer’s disease (AD). Various risk factors for Alzheimer’s disease, including aging, apolipoprotein E (APOE) inheritance, smoking, hypertension, obesity, diabetes, and alcohol consumption, contribute to increased oxidative stress. Oxidative stress increases the expression and activity of γ-secretase and beta-site APP cleaving enzyme 1 (BACE1) by activating double-stranded RNA-dependent protein kinase (PKR), followed by phosphorylation of eukaryotic translation initiation factor-2α (eIF2α) and activation of c-Jun N-terminal kinase (JNK), promoting Aβ formation. However, oxidative stress decreased the activity of α-secretase, which prevents Aβ formation. Lipid oxidation product 4-hydroxy-2-nonenal (HNE) can increase toxic Aβ and promote its tendency to aggregate. In addition, HNE can change the protein conformation, increase the bilayer fluidity, and disrupt the phospholipid asymmetry with externalization of phosphatidylserine (PS) of synaptosome membranes. Oxidative stress can oxidize low-density lipoprotein receptor-related protein 1 (LRP1), impairing its clearing function and causing extracellular Aβ accumulation. In addition, ROSs can promote the activity of glycogen synthase kinase-3β (GSK3β), p38 MAPK (mitogen-activated protein kinases), cyclin-dependent protein kinase-5 (CDK5) to cause tau hyperphosphorylation (p), leading to the formation of intracellular tangles.

AD is an age-related neurodegenerative disease. During the aging process, there is a decline in the antioxidant defense system and elevated ROS production, resulting in a progressive increase in oxidative stress (Figure 3). Mitochondrial dysfunction is a hallmark of aging. The functional decline of mitochondria during aging reduces the energy supply, causes cellular damage, and increases ROS production [78]. Increased activity of NADPH oxidase with aging and other free-radical generating sources also contributes to age-related oxidative stress [79].

Inheritance of apolipoprotein E4 (APOE4) is a major risk factor for the development of AD. APOE can form lipoproteins by binding with lipids, regulating the packaging and transport of cholesterol and other lipids through the bloodstream. APOE4 loses the role of APOE in lipid homeostasis. APOE4 also disrupts cholesterol transport and impairs the ability of astrocytes to process Aβ [80]. The presence of the *APOE4* genotype increases the susceptibility to oxidative damage. Since APOE4 lacks cysteine residues, it is unable to bind to and scavenge lipid peroxidation product HNE, so HNE can continue to damage cellular components and cause neuronal death [81].

Obesity has been closely associated with cognitive impairment and is considered a risk factor for developing AD. Obesity is linked to increased generation of ROS in adipose and other tissues in multiple ways, including NADPH oxidase activation, oxidative phosphorylation, glyceraldehyde auto-oxidation, PKC activation, and polyol and HMP pathways [82]. In addition, obesity has been considered a chronic low-grade inflammatory status, and obese individuals have elevated proinflammatory cytokines, which can stimulate the generation of ROSs [83]. Moreover, obesity may result in an overload of supply in mitochondria for the Krebs cycle and an overactive mitochondrial respiratory chain, leading to impaired mitochondrial function and elevated ROS formation [84].

Uncontrolled hypertension has been associated with cognitive impairment and AD. High blood pressure can induce oxidative stress by promoting the generation of ROSs. High blood pressure can activate the receptor for advanced glycation end products (RAGE) to increase the Aβ deposition, which was prevented by inhibition of AGE formation and oxidative stress [85]. Activation of RAGE leads to the nuclear translocation of NF-kB by activating phosphatidylinositol-3-kinase (PI3K)/Akt, NADPH oxidase/ROS [86], Ras-MAPK kinase (MEK), and small Rho GTPase proteins [87]. NF-kB in the nucleus promotes the gene transcription of proinflammatory cytokines [88]. Oxidative stress reversely impairs blood vessel vasodilatory regulation and induces inflammation to injure the endothelial lining and promotes atherosclerosis, raising blood pressure [89].

Type 2 diabetes is a well-known risk factor for developing AD. Increased oxidative stress and induction of inflammation through aberrant glucose metabolism have been elucidated [28]. Chronic hyperglycemia causes excessive glucose to be metabolized, reducing NAD^+^ to NADH, which is oxidized in the mitochondria by mitochondrial electron transport chain complex 1, and superoxide ions are generated. The elevated superoxide ions and the induction of oxidative stress decrease the activity of GAPDH, resulting in subsequent inhibition of glycolysis and the switch of the metabolism of glucose products by HMP and polyol pathways, resulting in elevated generation of ROSs. Of course, hyperglycemia also activates AGEs/RAGE signaling pathways to promote the generation of ROSs and induction of an inflammatory response [90].

Chronic alcohol consumption can cause brain cell damage and increase the risk of AD [91]. Alcohol is primarily metabolized by alcohol dehydrogenase, converting alcohol to acetaldehyde, a toxic and reactive molecule. Then, acetaldehyde is metabolized by aldehyde dehydrogenase to form acetate. NADH is produced during these reactions, activating the respiratory chain reaction and consuming more oxygen, leading to an increased ROS formation. High levels of ethanol may be metabolized to acetaldehyde by CYP2E1. Alcohol consumption can induce the upregulation of CYP2E1. Superoxide anion radical and hydrogen peroxide are produced through CYP2E1 enzyme activity. In addition, alcohol decreases the activities of antioxidants, including SOD, catalase, and GPX [92].

Smoking during lifetime is associated with a significant increase in risk of developing AD, and cumulative cigarette exposure greatly increases this possibility [93,94]. Oxidative stress is obviously a great contributor of smoking to prompt the development of AD. Cigarette smoke contains a complex mixture of chemicals, including a host of ROSs. The gas and particulate phases of cigarette smoking have extremely high concentrations of ROSs and other oxidizing agents [95]. Smoking can diminish the endogenous antioxidants, such as vitamin E and glutathione, to lower the body’s antioxidant system [96]. Cigarette smoke triggers oxidative stress, causing lipid, protein, and DNA damage, stimulating inflammatory signaling cascades, leading to increased release of inflammatory cytokines [97].

### 4.3. Oxidative Stress and Extracellular Plaques

Extracellular plaques are composed of aggregated Aβ combined with redox-active metal ions, including copper, iron, and zinc, which can regulate the catalyzing process with the production of ROS. Significant increase in metal ions, copper and zinc in particular, was illustrated in AD brains. The metal–Aβ complex catalyzes a reductant and transfers an electron to oxygen, to produce superoxide anion, hydrogen peroxide, and the hydroxyl radical [98].

Oxidative stress increases the expression and activity of γ-secretase and beta-site APP cleaving enzyme 1 (BACE1), a rate-limiting enzyme for producing Aβ, by activating double-stranded RNA-dependent protein kinase (PKR) followed by phosphorylation of eukaryotic translation initiation factor-2α (eIF2α) and activation of c-Jun N-terminal kinase (JNK) [99]. BACE1 primarily cleaves APP, producing soluble fragment and a membrane-bound fragment, which is further processed by γ-secretase to produce Aβ peptides [100]. BACE1 activity correlated with toxic Aβ [101]. The aldehydic product of lipid peroxidation, HNE, induced up-regulation of BACE1, which is mediated by activating JNK and p38MAPK [102]. In contrast, oxidative stress decreased the activity of α-secretase [103], which cleaves APP within the Aβ domain in a non-amyloidogenic pathway, preventing Aβ aggregation (Figure 3).

The ROS oxidizes Aβ peptide and other cellular components to expand the oxidative damage [104]. Oxidation of a single methionine residue at position 35 of the Aβ was observed in the genetic APP model of Alzheimer’s disease in mice [105]. The residue methionine 35 can be oxidized to its sulfoxide or further to methionine sulfone. Knocking out methionine sulfoxide reductase in a human APP mouse model increased the level of soluble methionine sulfoxide Aβ [106]. The Aβ with oxidized methionine was also found in the brains of AD patients [107]. How methionine oxidation affects Aβ aggregation is complicated. In an in vitro experiment, the fibrillization of Aβ with methionine 35 oxidation induced by hydrogen peroxide was reduced compared to that of the regular peptide. However, no significant difference was found between regular fibrils and oxidized peptides under transmission electron microscopy [108]. Lipid oxidation product HNE can increase toxic Aβ and covalently modify the histidine side chains of Aβ to increase its affinity for lipid membranes and promote its tendency to aggregate [101,109]. In return, Aβ stimulates polyunsaturated lipids to promote the copper-mediated generation of HNE. Aβ residue tyrosine 10 has been considered a pivotal residue targeted by hydrogen peroxide in the presence of Cu (II) [110].

Oxidative stress can disrupt cellular clearing machinery, leading to Aβ accumulation. Low-density lipoprotein receptor-related protein 1 (LRP1) acts as a receptor for various ligands and internalizes these ligands by endocytosis into cells for processing or degradation. LRP1 plays a role in controlling the efflux of Aβ from the brain to the blood [111]. LRP1 oxidation impairs its clearing function, leading to an accumulation of Aβ [112].

### 4.4. Oxidative Stress and Intracellular Neurofibrillary Tangles

Oxidative stress interacts with protein kinases to induce the phosphorylation of the protein tau, which is an early sign of AD development. Tau binds to microtubules and stabilizes microtubules, which play an important role in neuronal structural stability and axonal transport. The hyperphosphorylation of tau leads to the loss of its ability to bind to microtubules, resulting in microtubule instability, aggregation, and subsequent formation of neurofibrillary tangles. The phosphorylation sites, including Ser262 and Ser356, are within the tubulin-binding domain, the phosphorylation of which inhibits the binding of normal tau to microtubules and thereby disrupts microtubule assembly. Hyperphosphorylated tau is the major component of the intracellular neurofibrillary tangle. Phosphorylation of tau at Ser262 has been identified as an early pathological change in AD that subsequently leads to the accumulation of tau. In contrast, inhibition of tau phosphorylation at Ser262 reduced tau toxicity and curbed tau-induced neurodegeneration [113]. Numerous phosphorylated sites of tau have been found with different contributions. Among them, Tyr18 and Thr231 tau phosphorylation have been shown to have a progressive increase with increasing neuropathological degree evaluated by Braak stages [114]. An increase in phosphorylation at Ser199, Ser202/Thr205, and Ser422 was only found in the late Braak stage of AD. Phosphorylation at sites Ser396-404 of tau protein at the carboxyl terminus was found as an early event in AD brains [115].

Several kinases are responsible for the phosphorylation of tau during oxidative stress. Glycogen synthase kinase-3β (GSK3β) is closely associated with and interacts with tau. Activated GSK-3β leads to the hyperphosphorylation of tau [116]. GSK-3β is one of the two isoforms of GSK-3 and is specifically expressed in the CNS. The main target of GSK-3β is β-catenin, which is a key signaling protein in the Wnt/GSK-3β/β-catenin pathway. β-catenin is a transcriptional co-activator and translocates to the nucleus with GSK-3β inhibition, promoting the transcription of Wnt target genes to protect cells by binding to T-cell factor/lymphoid enhancer-binding factor (TCF/LEF), CREB-binding protein, and/or p300 protein [117]. Without Wnt, unphosphorylated (activated) GSK-3β can phosphorylate β-catenin, leading to β-catenin degradation through the ubiquitin-proteasome system [118]. In addition, GSK3β can also inhibit the activation of Nrf2 to reduce the expression of antioxidant genes, resulting in increased oxidative stress in the nervous system [119]. The activity of GSK3β underlies its important pathogenic roles in AD and other neurodegenerative diseases. However, controversial results were obtained in rabbits. Intracisternal injection of aggregated Aβ induced GSK-3β nuclear translocation, Tau hyperphosphorylation, and oxidative mitochondrial mtDNA damage. GSK-3β inhibition with lithium did not prevent Tau hyperphosphorylation or mtDNA damage, although lithium prevented caspase activation, suggesting that nuclear translocation of GSK-3β may not be related to GSK-3β-induced tau phosphorylation [120]. However, the link between caspase activation and mtDNA damage has been established [121]. The results of the study should be followed up on and further solidify the relationship. In addition, the results also suggest that GSK-3β nuclear translocation might not play a dominant role in phosphorylating tau and in Aβ-induced AD pathogenesis in this setting, since other mechanisms may be involved (Figure 3).

MAPKs are another family of kinases for tau phosphorylation. Aβ-induced activation of p38MAPK that causes tau hyperphosphorylation is mediated by oxidative stress, since the antioxidant Trolox prevents Aβ-induced tau phosphorylation [122]. In addition, the induction of cysteines that form disulfide bonds in tau protein by oxidative stress promotes intracellular tau accumulation and toxicity [123].

Cyclin-dependent kinase 5 (CDK5) is also involved in tau phosphorylation and its regulation [124]. CDK5 normally phosphorylates tau to stabilize microtubules. Oxidative stress can activate CDK5 with aberrant high activity, causing neuronal apoptosis and tau hyperphosphorylation [125]. CDK5 is a complex that consists of the catalytic subunit and a polypeptide with a molecular mass of approximately 25 kDa (p25), which is a calpain-mediated cleavage fragment of p35. CDK5-p25 has a stronger capability than its full-length transcript to induce tau phosphorylation [126,127]

Microtubule-affinity-regulating kinases (MARKS) are a group of kinases that can phosphorylate microtubule-associated protein tau. Oxidative stress induced the activation of MARKs and hyperphosphorylation of tau in hydrogen peroxide-treated neuroblastoma cells. The activation of MARKs correlated with tau hyperphosphorylation at Ser262 on the microtubule-binding domain, a site that is essential to maintain microtubule stability and is the initial phosphorylation site in AD [114].

Although AMP-activated protein kinase (AMPK) can phosphorylate tau protein, the effects of AMPK activation in AD in complicated. AMPK activation may have an antioxidant role. Activated AMPK promotes Nrf2 translocation to the nucleus, where it binds to ARE and upregulates the expression of antioxidant proteins, including NADPH oxidase, HO-1, and SOD [128]. The downregulation of AMPK activity in AD has been observed, supporting its protective activity in AD [129]. AMPK is an important serine/threonine protein kinase that regulates the energy status of cells, and its activity is regulated by relative levels of AMP and ATP. Lower energy should induce the activation of AMPK; the inhibition of AMPK in AD, which has low ATP production, seems to be contradictory. It is possible that other factors or feedback-regulating mechanisms might exist in AD. Chronic oxidative stress, inflammation, and AD-associated pathogenesis may disrupt the sensing ability of AMPK to energy status and cause a decline in AMPK activity.

Casein Kinase 1 delta (CK1δ) has been shown to phosphorylate tau at specific AD-associated residues, including Ser68, Thr71, Ser214, and Ser289. It is an in vitro study, the results of which should be further reconfirmed in animal models and AD human brains. Nevertheless, the co-colocalization of CK1δ with tau in neurons and up-regulation of its expression were observed in the brain of AD patients [130].

Moreover, oxidative stress has been shown to inhibit tau dephosphorylation. Protein phosphatase 2A (PP2A) is a major phosphatase that dephosphorylates tau, preventing its aggregation and maintaining normal tau function. Oxidative stress can induce the translocation of endogenous inhibitor-2 of PP2A (I2PP2A) from the neuronal nucleus to the cytoplasm, where it inhibits PP2A activity, leading to tau hyperphosphorylation [131]. Peptidyl prolyl cis-trans isomerase 1 (Pin1) is another enzyme for tau dephosphorylation. The downregulation and oxidation of Pin1 have been observed in the AD hippocampus. The oxidation of Pin1 diminishes its activity, leading to the impairment of tau dephosphorylation and the degradation of APP, increasing tau phosphorylation and the accumulation of Aβ aggregates [132].

### 4.5. Oxidative Stress and Neuronal Dysfunction

AD is characterized by progressive memory loss due to impairment of synaptic plasticity. Loss of synapses in the hippocampus correlates best with cognitive impairment in AD patients. Synaptic degeneration occurs prior to the loss of neurons. Oxidative stress causes neuronal damage by interacting with cellular components. Oxidative stress-induced lipid peroxidation disrupts the neuronal and synaptosome membranes. The products of lipid peroxidation also interact with cellular proteins, changing their structure and functions. HNE has been shown to alter the protein conformation, increase the membrane bilayer fluidity, and disrupt the phospholipid asymmetry with externalization of phosphatidylserine (PS) of synaptosome membranes, leading to synaptic damage [133,134]. In addition, an increased expression of genes related to oxidative stress and DNA damage responses and elevated neuronal DNA double-stranded breaks were identified in intraneuronal Aβ burdened neurons, indicative of oxidative DNA damage, which occurred prior to the plaque formation [135].

The dysregulation of glucose metabolism is a common feature of AD due to the inhibition of enzymes in glucose metabolism by oxidative stress. In AD, a shift from aerobic metabolism to anaerobic glycolysis has been observed. Redox proteomics is a powerful tool that has identified oxidized proteins and has been applied to AD research. In the brains of AD, oxidation of multiple proteins that are involved in glucose metabolism has been identified [136,137] (Table 1). The reduced activities of these proteins or enzymes resulted in the loss of ATP and energy deficiency in the brain, impairing normal mitochondrial function, further inducing the generation of ROSs, and neurodegeneration.

Oxidative stress induces neuroinflammation, further causing neuronal damage. There exists plaque-dependent and -independent induction of neuroinflammation in AD. In plaque-dependent inflammation, Aβ plaques activate microglia and astrocytes, releasing proinflammatory cytokines [138]. The plaque-independent inflammatory reaction is associated with increased levels of soluble and oligomeric Aβ, which are much more toxic amyloid species in the brain and stimulate neurons to produce cytokines. The neuron-derived inflammatory response in AD brains occurs prior to the formation of insoluble Aβ plaque and neurofibrillary tangles [139], implying that it might be involved in the early development of AD.

Mitochondrial dysfunction is a common feature of AD and a cause of neuronal cell death. The dysfunction of mitochondria causes ATP deficiency and exaggerates ROS production, leading to the expansion of oxidative stress and subsequent induction of neuroinflammation. Oxidative stress also impairs mitophagy, losing its ability to degrade defective mitochondria, and causes a gradual accumulation of defective mitochondria in neurons, which has been implicated in AD [140]. In addition, mitochondrial dysfunction causes the deposition of β-amyloid plaques in mitochondria, further impairing the normal mitochondrial anterograde and retrograde transport in hippocampal neurons, resulting in synaptic degeneration in AD brains [141].

**Table 1:** Oxidized proteins involved in energy metabolism and stress in AD brains.

Proteins	Function	Role in AD	References
ATP synthase	ATP synthase is mitochondrial complex V of the electron transport chain that plays a key role in ATP production.	The oxidation of its alpha-subunit has been found in the AD brain. Decreased activity of ATP synthase was observed in the cortex of an AD mouse and patients.	[142,143,144]
Glyceraldehyde 3-phosphate dehydrogenase (GAPDH)	GAPDH is a key enzyme in glycolysis that metabolizes glucose to ATP.	Oxidative stress can induce GAPDH to denature and aggregate, decreasing its activity. Aggregated GADPH promotes amyloidogenesis. Oxidative stress also causes the interaction between GADPH and c-Jun N-terminal kinase (JNK) and activates JNK.	[145,146,147]
Enolase 1 (ENO1, alpha-enolase)	ENO1 is a glycolytic enzyme catalyzing the conversion of 2-phosphoglycerate to phosphoenolpyruvate.	ENO1 is upregulated in AD, reflecting a defense mechanism. ENO1 also interacts with heat shock protein 70 (HSP70) to protect neurons against oxidative stress. The ENO1 oxidation and decreased activity were found in AD brains.	[136,137,147,148]
Phosphoglucomutase 1 (PGM1)	Glycolytic enzyme catalyzes the conversion between glucose-1-phosphate and glucose-6-phosphate	The oxidized PGM1 and decreased activity were found in late AD patients.	[149]
Fructose bisphosphate aldolase (FBA)	Converts fructose 1,6-bisphosphate to glyceraldehyde 3-phosphate and dihydroxyacetone phosphate in glycolysis. It also plays a role in DNA repair.	Oxidative stress can inactivate FBA in AD brains, disrupting glucose metabolism, reducing ATP production, impairing DNA repair, and causing neuronal dysfunction.	[150,151]
Creatine kinase (CK)	Facilitates the transfer of phosphate between ATP and creatine, increasing ATP generation from ADP	Cytosolic brain-type creatine kinase (BB-CK) was significantly inactivated by oxidation in AD patients, and carbonylated BB-CK was identified in AD brains.	[152]
Phosphoglycerate mutase 1 (PGAM1)	A crucial enzyme in glycolysis, which catalyzes the interconversion of 3-phosphoglycerate and 2-phosphoglycerate	The oxidation of PGAM1 was observed in AD, altering its structure and function and impairing its role in energy metabolism.	[153]
Pyruvate kinase	Glycolytic enzyme, catalyzing the transfer of phosphate from phosphoenolpyruvate to ADP, producing pyruvate and ATP.	Pyruvate kinase M2 (PKM2) is an activator of γ-secretase, which is involved in the production of Aβ. ROS inhibits PKM2, potentially increasing Aβ production and impairing energy production.	[154,155]
Lactate dehydrogenase (LDH)	Catalyzes the interconversion of pyruvate and lactate, playing a role in glycolysis and ATP production.	Increased lactate levels and the activity of LDH were considered a promoting factor in Aβ accumulation and plaque formation. Oxidative stress may induce cells to shift more to glycolysis and increase LDH.	[156,157]
Malate dehydrogenase (MDH)	Catalyzes the oxidation of malate to oxaloacetate using the reduction in NAD^+^ to NADH in the tricarboxylic acid cycle and the malate-aspartate shuttle.	Oxidative stress can increase mitochondrial MDH activity and mRNA levels in hippocampal cells, possibly as a compensatory response.	[158]
Triose-phosphate isomerase (TPI)	Catalyzes the conversion of dihydroxyacetone phosphate to glyceraldehyde-3-phosphate in glycolysis.	In the AD brains, TPI is oxidized by carbonyl and nitrotyrosinated modification, disrupting its enzymatic activity. Nitrotyrosinated TPI interacts with Tau to promote intraneuronal aggregation and increases methylglyoxal toxic proteins.	[142,159]
Ubiquitin Carboxyl-terminal Hydrolase L1 (UCH-L1)	Deubiquitinates proteins, influencing their degradation pathways.	Oxidative stress induces the oxidation of UCH-L1, decreasing its activity and impairing protein quality control, which is implicated in AD pathology. In AD, a reduction in UCH-L1 levels is observed.	[160,161]
Glucose-regulated protein precursor (GRP)	GRP78 and GRP94 are primarily expressed in the ER lumen to regulate the proper folding of proteins synthesized within the endoplasmic reticulum (ER).	The ER stress and oxidation of GRP78 and GRP94 have been observed in AD brains, leading to misfolding of proteins and aggregation.	[162,163]
Superoxide dismutase (SOD)	Catalyzes the dismutation of the superoxide anion radical into normal molecular oxygen and hydrogen peroxide.	Increased expression of SOD1 and SOD2 within senile plaques, but decreased activities of SOD were found in AD. Another study indicates that levels of SOD1 were significantly decreased in human AD patients.	[164,165]
Glutathione peroxidase (GPX)	Catalyzes the reduction in hydrogen peroxide and lipid peroxides.	Knockout of GPX1 aggravates Aβ-induced neurotoxicity in cortical neurons. Increasing the expression and activity of GPX4 ameliorates cognitive function in AD.	[166,167]
Heme oxygenase-1 (HO-1)	Catalyzes heme into carbon monoxide (CO), free iron, and biliverdin, then to bilirubin.	Protects against oxidative stress and inflammation through its catalyzed products and also inhibits Aβ-induced neurotoxicity by CO.	[168]
Heat shock proteins (HSPs)	Functions in protein folding, degradation, and cellular stress responses.	HSPs may function to protect against oxidative stress and improve the clearance of Aβ plaques.	[169]
Glutamine Synthetase	Synthesizes glutamine from glutamate and ammonia, playing a role in ammonia detoxification and neurotransmitter regulation in the brain	Reduced expression and activity in AD brains have been observed.	[170,171]

### 4.6. Antioxidants in AD

Given the roles of oxidative stress in AD, antioxidants should have therapeutic effects on AD. Antioxidant supplements, including probiotics, selenium, melatonin, resveratrol, rosmarinic acid, carotenoids, curcumin, vitamin E, and coenzyme Q, have been investigated in AD. However, the efficacy was limited, and no benefit was found [172].

The limitation of the positive effects of these antioxidants has been attributed to short trial durations (several weeks to 1 year), small sample sizes (fewer than 100 participants, most fewer than 50), and a lack of diversity among study participants (no study with multiple centers or worldwide involvement). In addition, the limited permeability of some antioxidants across the blood–brain barrier may also be an unfavorable factor. For example, some polyphenols like quercetin, curcumin, and resveratrol have lower bioavailability to cross the blood–brain barrier due to their poor absorption and rapid metabolism [173].

In the future, large-scale studies with extensive cooperation are required to determine the efficacy of antioxidants in AD. It is also possible that earlier use of antioxidants would be more effective, so subgrouping subjects based on the stages of AD should be considered for the trials. Since the complexity of the etiology in AD, combined therapy should be preferred.

## 5. Role of Oxidative Stress in Parkinson’s Disease

Parkinson’s disease (PD) is the second most prevalent neurodegenerative disease worldwide after AD. The primary pathological basis is the generation of the dopaminergic neurons in the Substantia nigra with the accumulation of Lewy bodies in the cytoplasm of dopaminergic neurons. Lewy bodies are intraneuronal inclusions composed of misfolded and aggregated α-synuclein and other components, such as neurofilaments, ubiquitin, etc. Oxidative stress has been implicated in dopaminergic degeneration. Postmortem investigations have demonstrated that oxidative stress-induced damage to cellular macromolecules in nigrostriatal dopaminergic neurons in the brains of PD patients. ROSs induction of lipid peroxidation, DNA damage, inflammation, and protein misfolding result in neuronal dysfunction and death in the Substantia nigra.

Multiple mechanisms have been associated with oxidative stress in the pathogenesis of PD. The risk factors for PD, such as aging, heavy metal exposure, and pesticides, may contribute to the induction of oxidative stress. The mutations of PD-associated genes play an important role in the vulnerability of dopamine neurons to oxidative stress [174].

### 5.1. Mitochondria and Oxidative Stress in PD

Mitochondrial dysfunction with oxidative stress has been considered a key pathological mechanism of PD. Impaired electron transport chain, altered mitochondrial dynamics, and disruption of mtDNA have been observed in PD. Post-mortem examination has demonstrated that the dysfunction of mitochondrial complex I contribute to the disruption of mtDNA integrity in the Substantia nigra [175].

**Figure 4:**
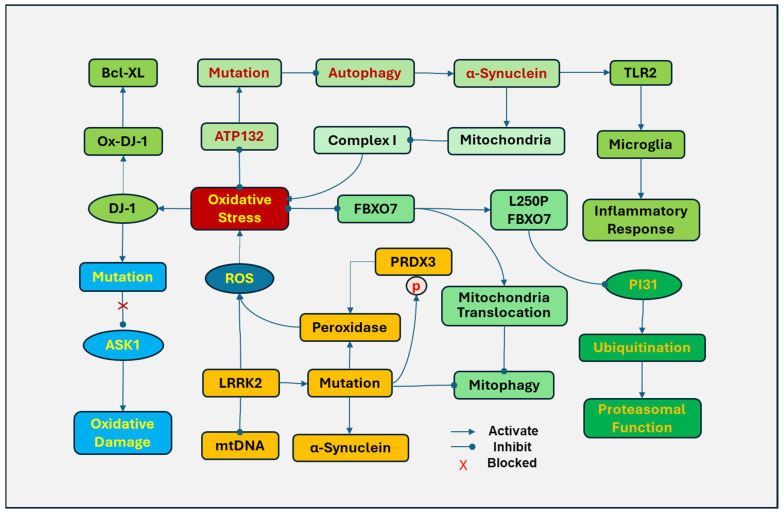
Associated gene mutations, mitochondria, and oxidative stress in Parkinson’s disease (PD). DJ-1 (Parkinson’s disease protein 7, PARK7) is translocated to mitochondria in response to oxidative stress. Oxidized DJ-1 (Ox-DJ-1) protects cells by binding to Bcl-XL. Oxidative stress induced a mutation of DJ-1 that prevents it from being oxidized by inhibiting apoptosis signal-regulating kinase 1 (ASK1). The mutation of leucine-rich repeat kinase 2 (LRRK2) in mitochondria can disrupt mitochondrial DNA (mtDNA), increase the generation of ROS, and promote the translocation of dynamin-related protein 1 (DRP-1) to mitochondria to induce abnormal mitochondrial fission. LRRk2 mutations disrupt mitophagy and increase α-synuclein fibrils. In addition, mutations in the LRRK2 kinase domain upregulate phosphorylation of peroxiredoxin 3 (PRDX3), which reduces the peroxidase activity and increases ROS production. F-box only protein 7 (FBXO7) is involved in mitochondrial function, cell cycle, and proteasomal function by regulating ubiquitination. The *FBXO7* gene L250P mutation attenuated the interaction of FBXO7 with the proteasome inhibitor of 31,000 Da (PI31) and significantly reduced FBXO7 and PI31 levels in patient cells, impairing ubiquitination. Cells with the FBXO7 mutation also demonstrated disrupted mitochondrial function and mitophagy. ATP13A2 (PARK9), a lysosomal ATPase, inhibits oxidative stress in mitochondria by regulating endo-lysosomal polyamine export. The mutations in the *ATP13A2* disrupt autophagy, causing accumulation and aggregation of α-synuclein and accumulation of mitochondrial fragments in mitochondria. The aggregated α-synuclein in the inner mitochondrial membrane of dopamine neurons downregulates the activity of complex I and increases the generation of ROSs. In addition, extracellular oligomeric α-synuclein released from neuronal cells can activate toll-like receptor (TLR)2, leading to the activation of microglia and inflammatory responses.

The PD-related mutations of some genes are associated with oxidative stress and mitochondrial dysfunction (Figure 4). DJ-1 (Parkinson’s disease protein 7, PARK7) is sensitive to oxidative stress and plays a role in antioxidative defense. Under oxidative stress, DJ-1 is translocated to mitochondria, and the levels of oxidized DJ-1 in mitochondria are considered a potential biomarker for PD. Oxidized DJ-1 protects cells by binding to anti-apoptotic protein Bcl-XL and inhibiting its ubiquitination and degradation [176]. In addition, DJ-1 mutations have been implicated in familial PD. Oxidative stress preferably induces the mutation of DJ-1 at Cys106 in DJ-1, which loses the ability to be oxidized [176]. Mutations in DJ-1 have been linked to the development of recessively inherited early-onset PD [177]. DJ-1 also protects cells against oxidation damage by activating the ERK1/2 pathway and inhibiting apoptosis signal-regulating kinase 1 (ASK1) [178]. DJ-1 with a mutation at Cys106 causes the loss of its ability to inhibit ASK1 and to protect cell [179]. However, the exact role of this mutation in PD remains to be determined.

The mutation of leucine-rich repeat kinase 2 (LRRK2) is a common genetic cause in PD patients [180]. Overexpression of LRRK2 in mitochondria disrupts mtDNA with increased generation of ROSs. LRRK2 facilitates the translocation of dynamin-related protein 1 (DRP-1) to mitochondria to induce abnormal fission of mitochondria [181]. In contrast, inhibiting LRRK2 kinase activity protects cells against mitochondrial dysfunction [182]. LRRk2 mutations result in impaired mitochondrial function, disrupt mitophagy, and increase the susceptibility of dopaminergic neurons to PD-inducing reagents, including MPP^+^_,_ 6-OHDA, and ROSs [183]. LRRK2 mutations have been demonstrated to increase α-synuclein fibrils, reduce dopamine release, damage neurites, and induce neuronal death [184]. In post-mortem brains, mutations in the LRRK2 kinase domain were also found to upregulate phosphorylation of peroxiredoxin 3 (PRDX3), which reduces the peroxidase activity and increases ROS production [182]. The LRRK2 G2019S variant is the most common mutation of LRRK2 in PD [185].

F-box only protein 7 (FBXO7), also known as PARK15, is an adaptor protein in the SKP1–Cullin–1–F–box (SCF) E3 ligase complex, which is involved in various cellular processes, including mitochondrial function, cell cycle, and proteasomal function. Mutations in the *FBXO7* gene occur at an early onset, autosomal recessive familial PD, causing mitochondrial and proteasomal dysfunction by oxidative damage. The *FBXO7* gene L250P mutation attenuated the interaction of FBXO7 with proteasome inhibitor of 31,000 Da (PI31), a proteasome-binding protein, and significantly reduced FBXO7 and PI31 levels in patient cells, impairing the SCF ligase-mediated ubiquitination [186]. Cells with the FBXO7 mutation also demonstrated disrupted mitochondrial function and mitophagy. Oxidative stress induces FBXO to accumulate and form aggregates in mitochondria by promoting the translocation of FBXO7 to mitochondria and inhibiting mitophagy, leading to the disruption of mitochondria and the generation of ROS [187].

ATP13A2 (PARK9), a lysosomal ATPase, regulates active cation transportation across endosomal or lysosomal membranes. ATP13A2 inhibits oxidative stress in mitochondria by regulating endo-lysosomal polyamine export. ATP132-mediated polyamine transport activity was required to prevent mitochondrial toxins and rotenone-induced mitochondrial generation of ROSs [188]. The mutations in the *ATP13A2* gene result in lysosome dysfunction, impairing exocytosis and autophagic flux, which have been found in Kufor–Rakeb Syndrome, an early-onset atypical form of PD [189]. In addition, mutations of *ATP13A2* disrupt autophagy, causing aggregation of α-synuclein and accumulation of fragments in mitochondria [190,191]. The aggregated α-synuclein in the inner mitochondrial membrane of dopamine neurons downregulates the activity of mitochondrial complex I and increases the generation of ROSs [192]. In addition, extracellular oligomeric α-synuclein released from neuronal cells can activate TLR2, leading to the activation of microglia and inflammatory responses [193] and the impairment of the autophagy lysosomal pathway [194].

The aforementioned genes and mutations have been associated with mitochondrial dysfunction, oxidative stress, and neuroinflammation, which are involved in the pathogenesis of PD, implying that targeting their involved pathways will pave the way to finding novel therapeutic strategies for PD.

### 5.2. NADPH Oxidase and Oxidative Stress in PD

NADPH oxidase is one of the major sources of ROSs and has been implicated in the pathogenesis of PD. In 1-methyl-4-phenylpyridinium (MPP^+^) treated dopaminergic neurons, the generation of superoxide was prevented by NADPH oxidase inhibitors [195]. The expression of NOX1 was found in the nucleus of dopaminergic neurons in the substantia nigra of PD patients. NOX1 knockdown attenuated 6-hydroxydopamine-induced oxidative DNA damage and dopaminergic neuronal degeneration [196]. NOX in substantia nigra mediates paraquat-induced expression of α-synuclein and ubiquitin and α-synuclein aggregation [197].

Highly active NOX2 in neuronal and microglial cells in human PD and animal models was also observed. The aberrant NOX2 activity causes oxidative stress-related damage, leading to α-synuclein accumulation and mitochondrial protein import impairment in vitro in primary ventral midbrain neuronal cultures and in vivo in nigrostriatal neurons in rats [198]. Neuronal NOX2 activation was found in both chronic and acute phases of PD. However, microglial NOX2 activation was not found in acute and sub-acute PD models, suggesting microglial activation occurs later and plays a role in chronic disease. In addition, NOX2 has been shown to oxidize cysteine residues (30 and 289) in Ca^2+^/calmodulin-dependent protein kinase II α (CaMKIIα) and disrupt CaMKIIα/Ca^2+^/CaM complex, leading to intracellular Ca^2+^ accumulation and neuronal death [199].

The association of elevated LRRK2 kinase activity with elevated ROSs and lipid peroxidation was demonstrated in CRISPR-Cas9 gene-edited HEK293 cells, RAW264.7 macrophages, rat primary ventral midbrain cultures, and PD patient-derived lymphoblastoid cells [200]. LRRK2-induced cellular ROS production in PD may be mediated by NOX2 activity. In the study, LRRK2 was shown to phosphorylate Ser345 in the p47phox subunit of NOX2 and its subsequent translocation from the cytosol to the membrane-associated gp91phox subunit, leading to the activation of the NOX2 enzyme complex.

NOX4 has been contributing to dopaminergic neuronal death by inducing ferroptosis, which is characterized by iron accumulation, lipid peroxidation, and inflammation. NOX4 inhibition attenuated lipid peroxidation, reduced iron load in the substantia nigra of the PD model animals, and decreased the death of dopaminergic neurons [201]. NOX4 induced dopaminergic neuronal ferroptosis by activating PKCα; while NOX4 promoted neuroinflammation by upregulating astrocytic lipocalin-2 (LCN2). This study only investigated MTPT animal models. In addition, NOX4 may initiate other signaling pathways, which should be further explored. LCN2 is a secretory protein mainly from activated astrocytes, triggers inflammation by activating TLR4/NF-kB and JAK-STAT signaling pathways and increases NOX2 expression and ROS production [202].

### 5.3. Nrf2-ARE-NLRP3 Inflammasomes

Nrf2 is a redox-sensitive transcription factor that binds to ARE of its target genes. Nrf2 upregulates the production of antioxidant enzymes, including glutathione, SOD, HO-1, NADPH oxidase, and NQO1 [203]. It also inhibits the activation of NF-ĸB transcriptional activity and NLRP3 inflammasome, preventing the activation of proinflammatory cytokines [50,63,64]. NLRP3 inflammasomes induce the release of inflammatory cytokines, which can damage mitochondria and disrupt mitophagy, resulting in mitochondrial dysfunction and further aggravating oxidative stress, forming a vicious cycle that has been implicated in PD [204].

The decrease in the activity of Nrf2 with age contributes to PD. Under physiological conditions, Nrf2 is dominantly present in the cytosol of dopaminergic neurons, but elevated expression in the nucleus was observed in PD patients [174,205], which may upregulate the transcription of antioxidant genes. The expression of NQO1 levels is increased in the early and intermediate stages of PD, which may reflect a protective response; however, it is markedly diminished in the end-stage PD, losing its oxidative defense [206]. These investigations suggest that Nrf2 activity is upregulated to exert protection in response to oxidative stress at the early stage of PD. The downregulation of Nrf2 seemed to occur at the late stage of PD, losing its protective ability. Boosting the activity of Nrf2 should be considered a therapeutic strategy for PD [207].

### 5.4. Antioxidants in PD

Current mainstream treatment of PD focuses on increasing the level of dopamine and improving the dopaminergic transmission. The elucidation of the mechanisms underlying dopamine neuron damage gives ways to develop novel therapeutic strategies. In particular, oxidative stress has extensive effects on the pathogenesis of PD, influencing many aspects of the mechanisms that lead to the development of PD. Targeting the pathways that are involved in oxidative stress should possess effective therapeutic potential for PD.

Although numerous antioxidants have been successfully demonstrated to be effective in animal models of PD, their benefits in clinical trials were limited. However, the current outcomes cannot exclude the potential efficacy of antioxidants since the clinical subjects are pathologically complicated, and condition control is difficult. It is also possible that less effect is due to the late phase of participants when the oxidative damage has already been formed. Early diagnosis and the use of antioxidants in future trials should be considered.

## 6. Role of Oxidative Stress in Huntington’s Disease

Huntington’s disease (HD) is an autosomal dominant neurodegenerative disease characterized by the impairment of involuntary movement and cognitive impairment. Selective loss of neurons in the basal ganglia and cerebral cortex is one of the anatomical hallmarks of HD. The key pathogenic feature of HD is an abnormal expansion of CAG repeats in exon 1 of the *huntingtin* gene, producing a defective Huntingtin protein with a toxic functional gain to target transcription factors related to oxidative stress and inflammation. Solid evidence with the involvement of oxidative stress has been reviewed previously [208,209].

Mitochondria are the key source of ROSs in HD. In HD brains, decreased activities of complex II, III, and IV, impaired mitochondrial Ca^2+^, and disputed mitochondrial dynamics were observed [210]. Huntingtin protein mutant (mHTT) impairs the peroxisome proliferator-activated receptor (PPAR)-γ coactivator-1α (PGC-1α), which inhibits oxidative stress by inducing mitochondrial uncoupling proteins and promoting the expression of mitochondrial antioxidant enzymes, including SOD and GPX [211]. mHTT stimulates extrasynaptic N-methyl-D-aspartate-type glutamate receptor (NMDAR) and disrupts cyclic AMP response element-binding protein (CREB)-PGC-1α cascade [212], leading to impaired antioxidant enzyme activity. In greater detail, mHTT intervenes with transcriptional activation of CREB and TATA-box binding protein-associated factor 4 (TAF4), reducing PGC-1α expression, impairing axonal transport of mitochondria, and increasing the generation of ROSs [213]. In addition, mHTT disrupts mitochondrial trafficking and the balance between fission and fusion, reducing ATP and impairing bioenergetics [214], which has been associated with increased dynamin-related protein 1 (DRP1) and mitochondrial fission 1 (FIS1), and decreased levels of mitofusin 1 and 2 (MFN 1 and 2) and the optic atrophy 1 (OPA1) observed in the striatum and cortex of HD patients [215,216]. This results in the impairment of mitophagy, leading to dysfunctional mitochondria accumulation, disruption of bioenergetics, increased oxidative stress, and neurodegeneration. Moreover, mHTT in the mitochondrial intermembrane space can bind to TIM23, a subunit of the translocase of the inner membrane (TIM) complex, to inhibit protein import and disrupt mitochondrial proteostasis, leading to neuronal death [217]. The phosphorylation of mHTT at Ser 13 and Ser 16 at its N-terminal region decreases mHTT mitochondrial localization, inhibits its binding to TIM23, and improves mHTT-induced mitochondrial and neuronal toxicity [218]. The results support that the interaction between mHTT and TIM23 plays an important role in HD.

Abnormal polyglutamine expansions in HD cause the accumulation of misfolded proteins in the endoplasmic reticulum (ER), resulting in disruption of ER homeostasis and ER stress [219]. During the protein folding process, increased ROSs are generated as by-products, further disrupting the protein folding process [220].

Antioxidants targeting Nrf-2/ARE and PGC-1α have been demonstrated to be effective in slowing disease progression in animal models (rats and mice) of HD, showing decreased oxidative stress, lowered inflammatory response, improved motor performance, and improved neuronal survival [221]. The outcomes of limited clinical trials are premature in terms of improvement in motor and cognitive functions, but they showed some promise [221]. The problem derived from the disparity between experimental and clinical studies is similar to AD and PD. An elaborate design may improve the efficacy of antioxidants in HD. Improved delivery systems, such as nanoparticle-based carriers, liposome-packed articles, and viral vectors for gene therapy, may also provide a valuable tool to improve the effectiveness of antioxidants [222,223,224].

## 7. Role of Oxidative Stress in Amyotrophic Lateral Sclerosis

Amyotrophic lateral sclerosis (ALS), a disabling and fatal neurodegenerative disease, is characterized by selective loss of motor neurons in the motor cortex, brainstem, and spinal cord with progressive muscle weakness. Multiple lines of evidence indicate that an excessive generation of ROSs with impaired antioxidant defense represents an important pathological contributor to the loss of motor neurons in ALS [225]. The involvement of oxidative stress has been extensively investigated in post-mortem and animal models of ALS in both sporadic and familial ALS [226,227]. Oxidative stress induces the degeneration of the neuromuscular junction in ALS animal models, and increased sensitivity of the nerve terminal of neuromuscular junctions to ROS has been observed in these studies, leading to the impairment of neuromuscular transmission and weakness of muscles [228].

### 7.1. Gene Mutations, Mitochondria, and Oxidative Stress in ALS

Genetic variation that occurs in both sporadic and familial ALS is also linked to oxidative stress. The common mutated genes that are associated with ALS are SOD1, transactive response (TAR)-DNA binding protein (TARDBP, TDP43), angiogenin (ANG), fused in sarcoma RNA binding protein (FUS), and chromosome 9 open reading frame 72 (C9orf72). Their mutations induced pathogenesis, which has been associated with oxidative stress.

#### 7.1.1. SOD1

In the case of SOD1, approximately 2–3% of observed ALS cases and 20% of human familial ALS cases are attributable to a mutation in *SOD1*. *SOD1* was the first gene associated with ALS [229]. There are at least 200 pathogenic mutations in *SOD1* that have been identified in patients with ALS. Mutations in *SOD1* can result in its misfolding and aggregation, which lead to increased oxidative stress and mitochondrial dysfunction [230]. SOD1 mutations can acquire both gain of function detrimental to the cells and the loss of normal function.

The loss of SOD activity is one of the major pathogenic factors for SOD1 variants in ALS. SOD1 functions to convert superoxide radicals into hydrogen peroxide, which is then further turned into water and oxygen by peroxidase. SOD1 mutations can result in a build-up of hydrogen peroxide and cellular damage in motor neurons.

Some SOD1 mutants exhibit peroxidase activity, increasing the formation of highly reactive hydroxyl radicals. In yeast expressing SOD1 mutant (A4V, L38V, G93A, and G93C) exposed to hydrogen peroxide, a significant increase in the trapped radical was observed [231]. Increased peroxidase activity is also observed in mouse models overexpressing human SOD1 with AV4, H48Q, and G93A mutations, which can convert hydrogen peroxide into hydroxyl radicals, irreversibly inactivating the dismutase, aggravating the oxidative insults [232].

Mutations of SOD1 also impair mitochondrial function. ALS patients with the SOD1A4V mutation show significant increases in complex I and III activity of mitochondria in the motor cortex, leading to increased production of mitochondrial ROS [233]. The G93A mutation increases the susceptibility of motor neurons to oxidative stress, demonstrating more severe mitochondrial respiration failure and motor neuron death [234].

Other SOD1 mutations that have been linked to oxidative stress in ALS include G1H, G1L, A4V, G37R, H46R, H80R, G85R, D124V, D125H, E138Δ, and S134N. Motor neurons with these mutations either have lower antioxidant enzymes or have increased susceptibility to oxidative stress [235].

#### 7.1.2. TARDBP

TDP-43 is an RNA-binding protein predominantly expressed in the nucleus, but its cytoplasmic accumulation and aggregation are hallmarks of ALS. The TDP-43 proteinopathy has been associated with both familial and sporadic ALS [236]. TDP-43 can form aggregates combined with various proteins, including RNA binding proteins and mitochondrial components, to impair mitochondrial function and induce the generation of ROSs [237]. Wild-type and TDP-43 mutants have been shown to decrease mitochondrial membrane potential, reduce the activity of mitochondrial respiration complex I, and disrupt mitochondrial ATP synthesis. Oxidative stress also induces the formation of stress granules (SGs) in the cytoplasm and the accumulation of TDP-43 in SGs. In addition, oxidative stress can induce post-translational modifications, including phosphorylation, ubiquitination, and other changes, to enhance the aggregation of TDP-43 and impair the function of TDP-43 [236]. Oxidative stress further induces TDP-43 cytoplasmic translocation and aggregation in neurons, leading to the impairment of cellular function and neurodegeneration [238].

#### 7.1.3. *C9orf72*

Mutations in *C9orf72* with hexanucleotide repeat (GGGGCC) expansion (HRE) have been identified as the most common genetic cause of familial ALS. The progressive loss of locomotor performance in Drosophila models of C9orf72-ALS/FTD has been associated with mitochondrial dysfunction, impaired mitophagy, and increased generation of ROSs, which results from the activation of the Keap1/Nrf2 pathway [239]. The pathological outcomes of HRE in ALS have been recognized as the results of two mechanisms that include the toxic gain-of-function and loss-of-function of C9ORF72. The gain of toxicity results from RNA and dipeptide repeats (DPRs) [240]. DPRs, including poly-glycine-arginine (poly-GR) and poly-proline-arginine (poly-PR), induce oxidative stress in motor neurons and increase the stress granule assembly by activating c-Jun N-terminal kinase in motor neurons [241,242,243]. While poly-proline-alanine (poly-PA) enhances oxidative stress by decreasing the expression of Nrf2 and its target genes [244].

#### 7.1.4. FUS

The fused in sarcoma (FUS) protein plays a role in RNA metabolism and DNA repair. Its cytosolic accumulation in protein aggregates is also a hallmark of ALS. The FUS gene mutation can push its mislocalization to the cytoplasm and accumulation within SGs [245]. Mutant FUS loses its DNA ligating ability in response to oxidative stress. FUS mutants R521H and P525L have been identified to fail to repair oxidative stress-induced DNA damage, leading to the buildup of unrepaired DNA strand breaks in the motor neurons [246]. Overexpression of mutant FUS protein also resulted in an accumulation of damaged mtDNA and mitochondrial dysfunction [247].

#### 7.1.5. ANG

Angiogenin (ANG) is a ribonuclease that functions as an antioxidant defense mechanism in the nervous system. ANG can activate the Nrf2/ARE pathway in astrocytes to protect neurons against oxidant stress [248]. ANG mutants that lose the stability and ribonuclease activity of ANG have been associated with early ALS onset [249].

#### 7.1.6. CHCHD10

Mutations of coiled-coil-helix-coiled-coil-helix domain-containing protein 10 (CHCHD10) emerge as a mitochondrial protein involved in ALS. Mice with overexpression of CHCHD10 S55L mutants develop progressive motor deficits. CHCHD10 in aggregates induced a potent mitochondrial integrated stress response by activating mammalian target of rapamycin complex 1 (mTORC1) [250]. The mutation R15L in CHCHD10 of ALS patient fibroblasts impaired the stability of the protein, disrupting the assembly of mitochondrial respiration complex I [251]. CHCHD10 mutations lead to the impairment of the cristae organizing system complex assembly and oxidative phosphorylation in mitochondria. Overexpression of mutant CHCHD10 resulted in the loss of mitochondrial cristae and the loss of the ability to repair the disrupted mitochondrial genome induced by oxidative stress, leading to the accumulation of fragmented mtDNA molecules in patient muscle [252].

CHCHD2 is another mitochondrial intermembrane protein that forms a heterodimer with CHCHD10. Interestingly, the CHCHD2 P14L variant was found in ALS. The mutation of CHCHD2 promoted the mislocalization of CHCHD2 to the cytoplasm and elevated cytoplasmic Ca^2+^, facilitating the activation of calpain and caspase-3 and the aggregation of TDP-43 [253].

### 7.2. Oxidative and Inflammation Crosstalk in ALS

The interaction between oxidative stress and inflammation has been implicated in the pathogenesis of ALS. The surrounding glial cells and infiltrated immune cells are considered among the major producers of inflammatory cytokines and ROSs around motor neurons [254]. Oxidation products can stimulate the activation of microglia/macrophages to release inflammatory mediators. Carboxyethyl pyrrole (CEP) is a peroxidation product of polyunsaturated fatty acids, which showed an increased deposition in the ALS brains. The accumulation of CEP is dependent on activated inflammatory cells, since it is consistent with increased activities of myeloperoxidase (MPO) [255], a marker of neutrophils.

### 7.3. Toxin Exposure and Oxidative Stress in ALS

The environmental exposures and lifestyle factors that contribute to the development of ALS have also been linked to oxidative stress. The exposure to agricultural chemicals and heavy metals, military service, excessive physical exertion, chronic head trauma, and certain foods and smoking are involved in the pathogenesis of ALS by increased oxidative stress, which has been described in detail previously [256].

### 7.4. Antioxidants in ALS

Given the involvement of oxidative stress in the pathogenesis of ALS, antioxidants, such as vitamins C, E, selegiline, selenium, methionine acetylcysteine, and coenzyme Q10, have been studied as possible treatments. However, meta-analysis failed to show any clinical benefit in ALS patients [257]. The poor design and small group of subjects may be attributed to the unfavorable results. Nevertheless, the two FDA-approved drugs for ALS, riluzole and edaravone, have antioxidant properties. Riluzole blocks sodium channels in the brain and spinal cord, preventing calcium transport into nerve cells and excessive glutamate receptor stimulation. In an in vitro study, riluzole was shown to counteract the effects of hydrogen peroxide and reduce ROSs in SH-SY5Y cells [258]. However, riluzole was ineffective in SH-SY5Y cells with SOD1 G93A mutation, indicating that riluzole is effective in acute exposure to this drug, but not in chronic oxidative damage, which is consistent with its ineffectiveness on lifespan and motor performance in SOD1 G93A transgenic mice [259]. Edaravone functions as a free radical scavenger to reduce oxidative stress and slow the progression of ALS [260]. Edaravone is the first drug to prevent the motor function deterioration in early-stage ALS patients [261]. The outcomes of post-marketing studies for edaravone are not consistent. The study in Kuwait and Korea showed moderately beneficial effects in decreasing the progression of ALS. While studies in Italy and Israel failed to show any benefit [262]. Consequently, the effectiveness of antioxidant treatment for ALS is far from satisfactory. The insight into the mechanisms that are associated with oxidative stress, more specific patient recruitment, and more effective antioxidants should be considered in future clinical studies.

## 8. Conclusions

Generation of excessive ROSs and the induction of oxidative stress have been implicated in almost every aspect of pathogenic mechanisms in the development of AD, PD, HD, and ALS. ROSs cannot only directly damage proteins, lipids, DNA, and other cellular components, but they also stimulate an inflammatory response in the nervous system. In addition, the effectiveness of antioxidants in animal models of these diseases solidifies the deleterious effects of oxidative stress. However, inconsistent outcomes of antioxidants in the treatment of animal models and clinical subjects imply that insightful mechanisms should be further explored in terms of oxidative stress. Developing antioxidants with high bioavailability and easily accessible to the nervous system may improve the efficacy in the treatment of neurodegenerative diseases. In addition, the design of clinical trials for antioxidants should consider using a large-scale study with enough subjects, subdividing the subjects based on their disease progression, and delivering the drug through more efficient routes. Given the role of oxidative stress in the development of neurodegenerative diseases, antioxidants should hold great promise in the treatment of these diseases either as alternatives or monotherapy. The interconnected nature of oxidative stress and inflammation indicates that combined therapy with antioxidants and anti-inflammatory reagents may give more benefit to the neurodegenerative diseases.
